# Should We Perform Old-For-Old Kidney Transplantation during the COVID-19 Pandemic? The Risk for Post-Operative Intensive Stay

**DOI:** 10.3390/jcm9061835

**Published:** 2020-06-12

**Authors:** Philip Zeuschner, Urban Sester, Michael Stöckle, Matthias Saar, Ilias Zompolas, Nasrin El-Bandar, Lutz Liefeldt, Klemens Budde, Robert Öllinger, Paul Ritschl, Thorsten Schlomm, Janine Mihm, Frank Friedersdorff

**Affiliations:** 1 Department of Urology and Pediatric Urology, Saarland University, Kirrberger Street 100, 66421 Homburg/Saar, Germany; philip.zeuschner@uks.eu (P.Z.); michael.stoeckle@uks.eu (M.S.); matthias.saar@uks.eu (M.S.); 2 Department of Nephrology and Hypertension, Internal Medicine IV, Saarland University, Kirrberger Street 100, 66421 Homburg/Saar, Germany; urban.sester@uks.eu (U.S.); janine.mihm@uks.eu (J.M.); 3 Department of Urology, Charité-Universitätsmedizin Berlin, Corporate Member of Freie Universität Berlin, Humbold-Universität zu Berlin, and Berlin Institute of Health, Charitéplatz 1, 10117 Berlin, Germany; ilias.zompolas@charite.de (I.Z.); nasrin.el-bandar@charite.de (N.E.-B.); thorsten.schlomm@charite.de (T.S.); 4 Department of Nephrology, Charité-Universitätsmedizin Berlin, Corporate Member of Freie Universität Berlin, Humbold-Universität zu Berlin, and Berlin Institute of Health, Charitéplatz 1, 10117 Berlin, Germany; lutz.liefeldt@charite.de (L.L.); klemens.budde@charite.de (K.B.); 5 Department of Surgery, Campus Charité Mitte/Campus Virchow-Klinikum CCM/CVK, Charité-Universitätsmedizin Berlin, Corporate Member of Freie Universität Berlin, Humbold-Universität zu Berlin, and Berlin Institute of Health, Charitéplatz 1, 10117 Berlin, Germany; robert.oellinger@charite.de (R.Ö.); paul.ritschl@charite.de (P.R.)

**Keywords:** kidney transplantation, organ donation, deceased donor, Eurotransplant Senior Program, risk stratification, intensive care

## Abstract

Health care systems worldwide have been facing major challenges since the outbreak of the SARS-CoV-2 pandemic. Kidney transplantation (KT) has been tremendously affected due to limited personal protective equipment (PPE) and intensive care unit (ICU) capacities. To provide valid information on risk factors for ICU admission in a high-risk cohort of old kidney recipients from old donors in the Eurotransplant Senior Program (ESP), we retrospectively conducted a bi-centric analysis. Overall, 17 (16.2%) patients out of 105 KTs were admitted to the ICU. They had a lower BMI, and both coronary artery disease (CAD) and hypertensive nephropathy were more frequent. A risk model combining BMI, CAD and hypertensive nephropathy gained a sensitivity of 94.1% and a negative predictive value of 97.8%, rendering it a valuable search test, but with low specificity (51.1%). ICU admission also proved to be an excellent parameter identifying patients at risk for short patient and graft survivals. Patients admitted to the ICU had shorter patient (1-year 57% vs. 90%) and graft (5-year 49% vs. 77%) survival. To conclude, potential kidney recipients with a low BMI, CAD and hypertensive nephropathy should only be transplanted in the ESP in times of SARS-CoV-2 pandemic if the local health situation can provide sufficient ICU capacities.

## 1. Introduction

Health care systems all over the world have been facing major and unprecedented challenges since the outbreak of Coronavirus Disease 2019 (COVID-19). Extensive restrictions and nation-wide lockdowns were implemented to contain the spread of the novel coronavirus SARS-CoV-2. Its special features contributed to its fast and widespread transmission, including (1) being highly contagious, (2) the possible transmission from asymptomatic individuals and (3) causing mild symptoms in most of the infected patients [1,2]. Some countries were unexpectedly overwhelmed by a considerable increase in patients admitted to hospitals in need of intensive care [3]. Meanwhile, a worldwide shortage of personal protective equipment (PPE) in conjunction with limited bed capacities at intensive care units (ICU) resulted in suspension of elective surgeries. PPE and ICU beds were urgently needed as scarce medical resources for the management of COVID-19 cases and for the protection of the medical staff [4,5]. Another reason for postponing elective surgeries was the fear that patients admitted to hospital for elective surgery would become vectors for the transmission of a nosocomial infection with SARS-CoV-2 [3,4].

The outbreak of the pandemic also resulted in restrictions and cancellations in terms of kidney transplantation (KT) [6,7,8,9]. In Italy, a notable decrease in solid organ transplantation and procurement has already been observed in the first four weeks of the pandemic [10]. Currently, decisions on prioritizing certain procedures—including KT—are based on expert opinions rather than on evidence, contributing to different spread-dependent restrictions between regions [7]. In addition, it is unclear which immunosuppressive induction regimen can be administered safely. Especially, the administration of thymoglobulins causing long-lasting lymphopenia has been discussed critically, as a low lymphocyte count has been negatively associated with the disease severity of SARS-CoV-2 infection [1,11,12]. Even planned immunosuppression in living donation has been questioned [1]. The American Society of Transplantation and the European Association of Urology currently recommend to defer non-urgent KTs with living donors, but to perform urgent KTs—depending on the local situation [13,14]. However, the main aim should be rationing scarce medical resources, especially PPE, ventilators and ICU beds, while providing the best possible medical care to our patients [4].

The costs and benefits of a kidney transplantation during a pandemic should be counterbalanced [2]. We know that KT is the best treatment option for patients suffering from end-stage kidney disease (ESKD), with an improved survival rate and quality of life [15]. On the other hand, we lack information about the risk for admission to ICU after KT. In the context of scarce ICU resources, knowing about risk factors for ICU admission is crucial. Especially, older patients with comorbidities could have a higher risk for admission to ICU. The Eurotransplant Senior Program (ESP) is a special kidney transplant program which was initiated in 1999 to reduce waiting times by allocating kidneys from deceased donors aged ≥65 years to old recipients aged ≥65 years. Before that date, only 3% of patients aged 65 years or older actually received a KT offer within the Eurotransplant region, because younger patients with more favorable outcomes were prioritized [16]. In ESP, organ allocation is not based on immunological compatibility, but on local, regional or national allocation and AB0-compatibility, in order to reduce cold ischemia time (CIT). For this reason, risk assessment scores such as the Kidney Donor Risk Index (KDRI) are not integrated into the standard allocation protocols [17]. Double kidney allocation is not allowed at the beginning of the allocation procedure. Within the regular Eurotransplant Kidney Allocation System (ETKAS), kidneys can be allocated for donation after brain death (DCB) and, if allowed by national law, donation after cardiocirculatory death (DCD). Within the first 10 years, ESP has significantly increased the number of old kidney recipients. Local allocation resulted in shorter CITs and lower delayed graft function (DGF) rates compared to old kidney recipients in the regular Eurotransplant Kidney Allocation System (ETKAS) [18,19].

We lately had to decide whether or not to accept an allocated kidney from a 66 year old donor with a negative SARS-CoV-2 test result, allocated within ESP. The recipient was a 70 year old male with a solitary kidney who had an underlying hypertensive nephropathy. He had been on dialysis for 36 months and additionally suffered from coronary artery disease (CAD). This was the first organ offer within the ESP program at our department since the beginning of the SARS-CoV-2 pandemic. To provide valid information and thereby help decision-making in times of SARS-CoV-2, we conducted the first risk assessment for post-operative ICU stay among patients in the ESP so far. Additionally, the impact of an ICU admission on further outcome was assessed in this bi-center study.

## 2. Materials and Methods

In total, 105 KTs in the ESP performed at two tertiary referral centers were retrospectively analyzed. From 2010 to 2020, 40 (38.1%) and 65 (61.9%) kidneys were locally allocated to two transplant centers. In accordance with local law, all donors were brain-dead. No double kidney transplantations were included. All KTs were conducted in an open fashion by experienced transplant surgeons. After KT, the patients were admitted to an intermediate care unit by default. Only in the case of severe complications which could not be treated in an intermediate care unit, patients were admitted to the ICU. All kidney recipients received basiliximab as an induction treatment in combination with tacrolimus, mycophenolate mofetil and (methyl)prednisolone as the standard immunosuppressive regimen in both transplant centers.

This entire analysis was conducted in adherence with the correct scientific research work terms of the Charité Medical University of Berlin and Saarland University. Patients provided written informed consent and patient data was fully anonymized.

### 2.1. Data Collection and Outcome Measures

For the recipient characteristics, age, gender, BMI (kg/m^2^) and relevant health-conditions (arterial hypertension, CAD, diabetes mellitus, history of smoking) were obtained. The underlying cause for ESKD, duration and type of dialysis, and number of prior kidney transplantations characterized recipient’s nephrological history. For the graft characteristics, donor age, number of HLA-mismatches and cold ischemia time (CIT) were obtained. Regarding KT, operating time, warm ischemia time (WIT) and intraoperative complications served as surgical outcomes. Admission to ICU, length of ICU stay, complications based on Clavien Dindo within 30 days after surgery (major complications defined as ≥grade 3a) and length of hospital stay characterized the recipient’s postoperative course. The graft function was assessed by DGF rates, defined as the need for dialysis within 7 days after transplantation, and serum creatinine during follow-up. Over 10 years, graft and patient survival were compared.

As the primary outcome, risk factors for ICU admission after KT in ESP were identified. Therefore, patients with ICU admission were compared with patients without an ICU stay. To assess the influence of recipient and donor age on ICU admission, age-dependent comparisons were conducted, considering very old donors ≥75 years (very old-for-old vs. old-for-old) and very old recipients ≥70 years (old-for-very old vs. old-for-old). A multivariate binary logistic regression analysis identified significant risk predictors for ICU stay, which were used to create a risk model.

As the secondary outcome, the impact of ICU admission on further outcome was assessed. For this objective, survival and regression analyses identifying factors impacting graft and overall survival were calculated. Graft survival was always censored for death with functioning graft (DWFG).

### 2.2. Statistical Analysis

Categorical variables were reported as frequencies and proportions, and continuous data as the median and range. Fisher’s exact test and Mann-Whitney U test were conducted to compare between the groups. Kaplan Meier analyses compared graft and patient survival between groups by log-rank test. For binary logistic and cox regression analyses, covariates were included in multivariate regression analysis only if the respective effect was significant in the univariate analysis. For multivariate regression analyses, forward Wald selection was applied. The best cut-off for predicted probability of ICU stay in the multivariate risk model was estimated via ROC-analysis and Youden index. Statistical analyses were performed by SPSS version 25 with Fix pack 2 installed (IBM, Armonk, NY, USA). All tests were two-sided, and *p*-values < 0.05 were considered significant.

## 3. Results

### 3.1. Overall Results Regarding ICU Admission

Overall, 17 (16.2%) patients were admitted to the ICU for a median length of 2 days (range 1–27). The main reason for ICU admission was significant hypotension requiring catecholamines in the absence of acute bleeding in five (29.4%) patients. Three (17.6%) patients were admitted for respiratory insufficiency, three (17.6%) for sepsis with multiple organ failure, and two (11.7%) for cardiac infarction. One (5.9%) patient had hyperkalemia, another a compartment syndrome due to occlusion of iliac arteries. One (5.9%) patient had a significant bleeding requiring surgical re-exploration, another had his graft surgically removed because of arterial stenosis and consecutive graft necrosis, and required intensive care thereafter. The median time between KT and ICU admission was 0 days, as 10 (58.8%) patients were admitted to the ICU immediately after KT. In total, 4 (23.5%) patients were admitted on postoperative day (POD) 2 to 4, and 2 (11.7%) patients on POD 8 and 9. One (5.9%) patient was admitted to the ICU on POD 36; he suffered from a late-onset sepsis. The admission rate to the ICU did not differ between the two transplant centers.

Patients admitted to the ICU were insignificantly older than patients without an ICU stay (71 vs. 69 years, n.s.) (see Table 1). They had a lower BMI (24.2 vs. 26.7, *p* < 0.05) and CAD twice as often (64.7% vs. 35.2%, *p* < 0.05). Regarding the underlying renal disease, hypertensive nephropathy was more common in patients admitted to the ICU (35.3% vs. 10.2%, *p* < 0.05). In both groups, the median number of HLA-mismatches was four (range 1–6, n.s.). There was a tendency towards longer CIT for patients admitted to the ICU (667.8 vs. 552.3 min), but it was not significant.

Regarding KTs, patients admitted to the ICU had slightly longer operating times (212 vs. 180 min, *p* = 0.053), and neither WIT nor intraoperative complication rates differed (see Table 2). During the postoperative course, patients with an ICU stay suffered from more frequent and higher complications based on Clavien Dindo, although this was not significant. Although there were fewer minor complications, 9 (52.9%) patients admitted to the ICU had more complications at grade 5 (17.6% vs. 0, *p* < 0.01). Patients with an ICU stay were discharged insignificantly later (21.5 vs. 18 days, n.s.).

**Figure 1:**
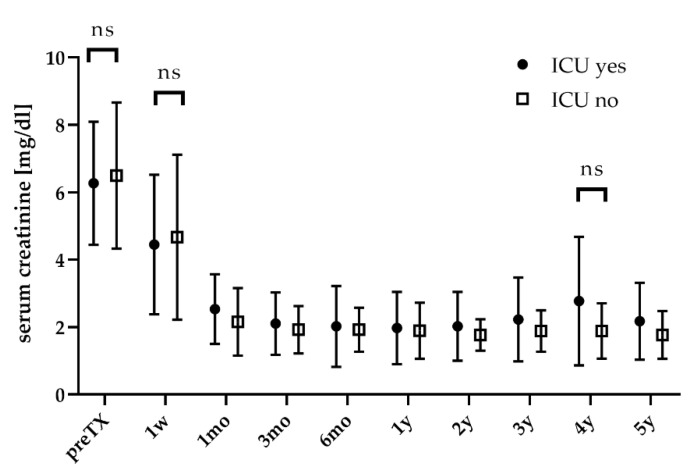
Graft function during follow-up. w: week; mo: month; y: year.

DGF rates were higher for patients admitted to the ICU (52.9% vs. 37.5%, n.s.) (see Table 2). Serum creatinine significantly declined after KT (*p* < 0.05) and did not differ between patients with or without ICU stay (see Figure 1).

### 3.2. Donor- and Recipient-Age-Dependent Comparison

In total, 28 (26.7%) patients received a graft from very old donors ≥75 years, compared to 77 (73.3%) old donors (‘old-for-old’) (see Table 3). When stratifying for donor age (very old-for-old vs. old-for-old), neither recipient nor graft characteristics differed. Grafts from very old donors had a tendency towards a longer CIT, which was not significant (677.1 vs. 540.6 min). Kidney recipients of very old donors had a tendency to be admitted to the ICU more frequently (21.4% vs. 14.3%, n.s.), but were discharged significantly earlier (16 vs. 20 days, *p* < 0.05). Neither DGF rates nor the kidney function differed during follow-up.

When stratifying for recipient age (old-for-very old vs. old-for-old), 47 (44.7%) recipients were ≥70 years old, and thereby were considered as very old (see Table 3). Regarding recipient characteristics, only the history of smoking differed, as fewer very old recipients had a history of smoking (8.5% vs. 24.1%, *p* < 0.05). Neither graft nor transplantation-specific factors were different. Very old recipients were admitted to the ICU insignificantly more often (21.3% vs. 12.1%). Graft function one week after KT was the only parameter that differed when comparing very old to old recipients, as very old recipients had a lower serum creatinine than old recipients (3.35 vs. 5.36, *p* < 0.01). During follow-up, the kidney function became equivalent.

### 3.3. Risk Model for ICU Stay

Among recipient and graft characteristics as well as transplantation-specific outcomes, the BMI of the recipient, an underlying hypertensive nephropathy and CAD were the only significant predictors for ICU admission in univariate and multivariate analysis (see Table 4). A higher BMI lowered the OR for ICU admission (OR 0.8, *p* < 0.01), but a hypertensive nephropathy (OR 4.0, *p* < 0.05) and CAD (OR 4.46, *p* < 0.05) significantly increased the OR for ICU admission during the hospital stay. Donor or recipient age did not impact the risk for ICU admission.

**Figure A1:**
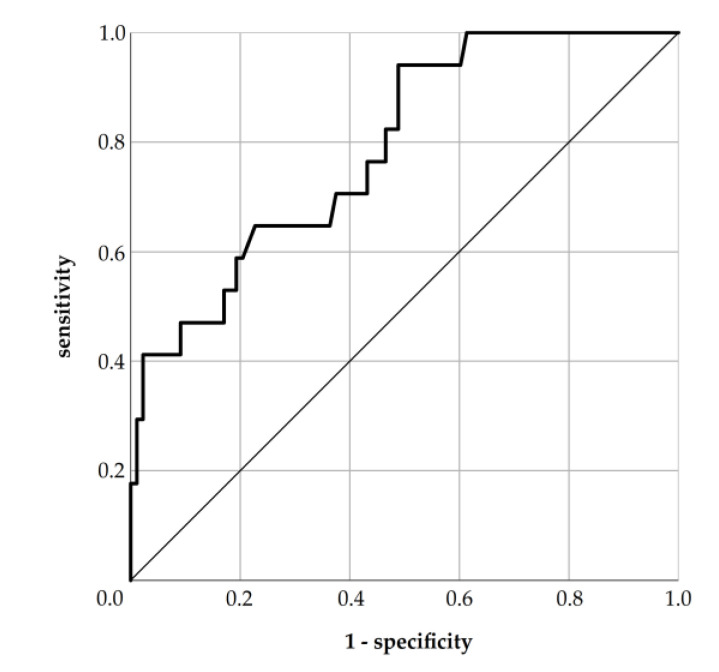
ROC analysis examining the relationship between the predicted probability of ICU stay and actual ICU admission.

When combining these three factors in a risk model to estimate the probability for ICU admission, the c-index reached 0.789 (*p* < 0.001) (see Figure A1). When setting the cut-off for the predicted probability of ICU admission to 0.08, which had highest Youden-index, the risk model reached a sensitivity of 94.1%, specificity of 51.1%, false positive rate (FPR) of 48.9%, false negative rate (FNR) of 5.9%, positive predictive value (PPV) of 27.1% and negative predictive value (NPV) of 97.8% (see Table A1).

### 3.4. Survival Analysis

For all 105 patients, the median length of follow-up was 49.5 months. The overall graft survival at 1, 5 and 9 years was 84%, 73% and 42%, respectively, with a median death-censored graft survival of 113.9 months. Median patient survival was 108.2 months, with a 1-, 5- and 9-year survival of 85%, 62% and 38%, respectively.

**Figure 2:**
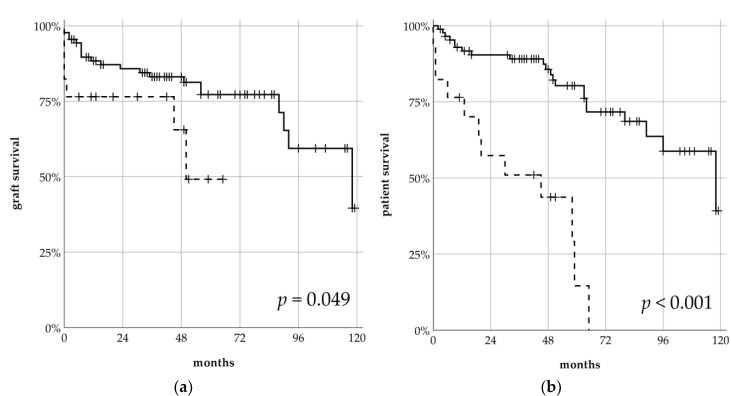
Death-censored graft survival (**a**) and patient survival (**b**) comparing patients admitted to the ICU (**dashed line**) vs. patients not admitted to the ICU (**solid line**) after kidney transplantation in the ESP program.

When stratifying for ICU admission, patients admitted to the ICU had a significantly shorter graft survival (59.1 vs. 115.7 months, *p* = 0.049) (see Figure 2a). Their 1- and 5-year graft survivals were 75% and 49%, and thereby worse compared to patients without an ICU stay (86% and 77%). Over the whole study period, the death-rate for patients with an ICU stay was almost three times higher compared to patients without an ICU stay (70.6% vs. 26.1%, *p* < 0.001). Consequently, the median patient survival for patients admitted to the ICU was significantly shorter (ICU 36.9 vs. 114.9 months, *p* < 0.001) (see Figure 2b). 1- and 5-year patient survival for patients admitted to an ICU was 57% and 0% and for patients without an ICU stay 90% (1 year), 72% (5 years) and 44% after 9 years, respectively. In total, 17 (48.6%) patients died with a functioning graft, and the DWFG rate did not differ between groups (ICU 50% vs. 47.8%, n.s.). Neither the age of the donor nor the recipient affected graft or patient survival (see Table A2).

In a multivariate cox regression, higher numbers of prior KTs and HLA-mismatches significantly shortened graft survival (hazard ratio (HR) for graft loss 9.66, *p* = 0.001; HR 1.53, *p* < 0.05) (see Table 5). Additionally, higher serum creatinine 1 month after KT was associated with worse graft survival (HR 1.37, *p* < 0.05). ICU admission during the hospital stay after KT did not affect graft survival. Regarding patient survival, a pre-transplant diabetes mellitus and an ICU admission during the hospital stay were significant predictors for worse outcomes in the multivariate analysis (HR for patient death 2.22, *p* < 0.05, HR 4.7, *p* < 0.001). Major complications during the hospital stay and the serum creatinine 1 month after KT were only associated with patient survival in univariate analysis.

## 4. Discussion

In this bi-centric study, an analysis of 105 kidney transplantations of deceased donors, allocated within the Eurotransplant Senior Program, was conducted. We aimed to identify risk factors for ICU admission after KT during a hospital stay in times of shortened PPE and ICU capacities because of the SARS-CoV-2 pandemic.

Overall, recipient and graft characteristics were comparable with other cohorts [19,20,21,22]. CIT was lower, lasting on average 9.5 h, while most other ESP programs have CITs averaging 10 to 12 h [19,20]. ESP aims to reduce CIT by prioritizing local organ allocation, as longer CITs have been clearly linked with higher DGF rates. Nonetheless, our DGF rate of 40% is higher than that of one of the largest ESP cohorts so far, with 1406 KTs, by Frei et al. They reported a median DGF rate of 29.7% [19]. In contrast, other groups have comparable DGF rates ranging between 34.7 to 41.1% in their ESP cohorts [22,23]. Chavalitdhamrong et al. even stated a DGF rate of 60.4% for 601 KTs, but for organs allocated by ECD (extended criteria donors) for donors aged 50–69 years, and 63.9% for donors aged ≥70 years [24].

In a high-risk cohort like ESP recipients, complications are common. There were 11.4% intraoperative complications, and 26.7% minor and 33.3% major complications occurred postoperatively, according to Clavien–Dindo. Reports on complication rates state highly variable results, mainly due to inconsistent definitions. Bentas et al. have “surgical complications” in 47% of cases in their ESP program, whereas Bahde et al. reported 15.7% intraoperative and 22.5% post-operative surgical complications among their recipients [23,25]. Only Gallinat et al. defined postoperative complications according to Clavien–Dindo. In their comparison of very old donors in the ECD program, the rate for major complications was 48%, defined as ≥grade 3b [26].

During follow-up, death-censored graft survival (1- and 5-year: 84% and 73%) and patient survival (1- and 5-year: 85% and 62%) were superior to Frei et al. and comparable with Quast et al., who retrospectively analyzed 217 ESP transplantations at their department from 1998 to 2014, considering donor age [19,20] (see Table 6). In accordance with Boesmueller and Giessing et al., the main reason for graft loss was death with functioning graft [18,22]. Our analysis comprises one of the longest follow-ups in ESP so far. Overall, graft-survival after 9 years was 42%, and patient survival was 38%. Quast et al. reported a 10-year patient survival of 40% for old donors, and 35% for very old donors, whereas graft survival was 30% and 10%, respectively.

Based on this data, we have identified risk factors for ICU admission during a hospital stay in the ESP. In times of the SARS-CoV-2 pandemic with a shortage of ICU capacities, risk stratification is crucial to identify patients at high risk for ICU admission (after KT). This aspect has rarely been addressed so far. To the best of our knowledge, only three working groups have stratified their data for ICU admission [27,28,29]. Two working groups focused on ICU admission at any time after KT, even years after KT, which clearly does not help when trying to decide whether or not to perform a KT during the present SARS-CoV-2 pandemic. Abrol et al. retrospectively analyzed 1527 kidney transplantations between 2007 and 2016 and found higher age, increasing BMI, pre-transplant dialysis and deceased donor transplantation to be associated with ICU admission in their multivariate analysis. Living donor KT and preemptive KT were associated with a lower risk [27]. Nonetheless, 82.8% of the included KTs were living kidney transplantations. As such, we are the first to report on the risk for ICU admission immediately after kidney transplantation in the ESP.

17 (16.2%) patients in our cohort were admitted to the ICU for a mean time of 2 days. More than 80% of patients were admitted directly postoperatively or within four days after KT. The main cause for ICU admission was significant hypotension requiring catecholamines. Overall, patients admitted to the ICU had a lower BMI, and CAD as well as hypertensive nephropathy were more common. Graft characteristics and surgical outcomes during transplantation did not differ. The DGF rate of patients admitted to the ICU was high, with 52.9%, but did not significantly differ from patients without an ICU stay (37.5%).

As stated elsewhere, neither the donor nor the recipient’s age had an impact on the postoperative course [18,20]. Therefore, age did not affect ICU admission rates in the regression analysis. We assume that within this (very) old patient cohort, age differences were not as important as in younger patient cohorts due to preselection during the workup for listing. As patients admitted to the ICU had a lower BMI, an increasing BMI lowered the risk for ICU admission (OR 0.8, see Table 4). This is an interesting finding, referring to the ‘obesity paradox’, which describes the association of obesity with higher mortality in the general population on the one hand, but with a survival advantage among obese patients with several diseases on the other hand. In this regard, meta-analyses have shown that patients with a higher BMI might have (i) a reduced risk of ICU admission or death when suffering from pneumonia, (ii) a reduced adjusted mortality when admitted to the ICU with sepsis, severe sepsis or shock, and (iii) a lower mortality on mechanical ventilation in an ICU [30,31,32]. Although the concept of the obesity paradox has been questioned, there is also convincing evidence for underlying molecular mechanisms, i.e., that a lower energy reservoir in underweight patients cannot equally counteract the adverse influence of increased catabolic stress [33,34].

As further variables, hypertensive nephropathy and CAD increased the OR for ICU admission by 4 and 4.5, respectively. Most patients were admitted to the ICU because of hypotension as a major symptom for cardiac insufficiency, which is more likely in patients with CAD. In addition, hypertensive nephropathy has been linked with a higher risk for cardiovascular events and death [35]. When combining these three independent risk factors in a risk model, it gained a c-index of 0.789 with a sensitivity of 94.1%, a FNR of 5.9% and a NPV of 97.8% (see Table A1). For this reason, our risk model is highly valuable for the identification of patients at high risk for ICU admission. When applied to our cohort, the risk model was false negative in only one case. We are aware that it has a rather low specificity and PPV, whereas the FPR is high. Furthermore, the confidence intervals for the corresponding odds ratios are large, because only 17 (16.2%) patients were admitted to the ICU and not all of them suffered from hypertensive nephropathy or CAD (see Table 1). However, the high sensitivity and NPV of more than 94% render our risk model an ideal search test.

Our patient who had an organ offer in ESP during the SARS-CoV-2 pandemic had a probability of 92.8% to be admitted to the ICU according to our risk model, with a hypertensive nephropathy, CAD and BMI of 29.4 kg/m^2^ (see A1 for further explanation). Of note, this patient was not included within the analyzed cohort. Indeed, after transplantation, he had to be admitted to the ICU on postoperative day seven due to urosepsis and suspected cardiac infarction. Infectious complications are common among old kidney recipients and have been shown to be their second most frequent cause for DWFG [16]. In our cohort, 3 out of 17 (17.6%) patients had to be admitted to the ICU because of sepsis. Especially in the context of the ongoing SARS-CoV-2 pandemic, the question of how to manage immunosuppression for KT recipients is still a matter of debate [11,12].

As standard, all patients were administered tacrolimus, mycophenolate mofetil, (methyl)prednisolone and basiliximab for induction therapy. Consequently, the regimen did not affect ICU admission rates. Since lymphopenia has been associated both with a higher risk for SARS-CoV-2 infection and for severe forms of Covid-19, the questions (i) whether or not to perform the transplantation at all and (ii) whether the induction therapy should be reduced were intensively discussed at the transplant center which had an organ offer in ESP during the SARS-CoV-2 pandemic [11]. Finally, the patient was transplanted and received an induction therapy with basiliximab, and unfortunately suffered from sepsis and neutropenia. For this reason, mycophenolate mofetil was stopped and the dose of prednisolone reduced. Of note, SARS-CoV-2 had been ruled out prior to transplantation and after the onset of sepsis again; as we have not experienced a major shortage of ICU capacities, we could guarantee maximum care for this patient at all times. However, we might decide differently if we receive another organ offer in the ESP program during the ongoing SARS-CoV-2 pandemic again.

Interestingly, ICU admission also proved to be an excellent indicator for the identification of patients at risk for short graft and patient survival. In Kaplan–Meier analysis, patients admitted to the ICU had a significantly shorter graft survival of 59.1 months; all of them died within five years (see Figure 2). Consequently, ICU admission impacted patient survival with a HR of 4.72, but did not impact graft survival in Cox regression (see Table 5). Diabetes mellitus was the only other covariate impacting patient survival. Other studies were inconclusive about the effect of pre-transplant diabetes mellitus or new-onset diabetes mellitus (NODAT) on patient survival. Some studies have found associations with NODAT, but not pre-transplant diabetes, with mortality and graft failure, and others the inverse [36,37,38]. By contrast, ICU admission did not impact death-censored graft survival in Cox regression. The individual number of kidney transplantations per patient (HR 9.66), number of HLA-mismatches (HR 1.53) and the serum creatinine one month after transplantation (HR 1.37) were significant. The negative impact of increasing HLA-mismatches on graft survival was reported more than two decades ago [39]. To shorten waiting times for old recipients, ESP does not integrate HLA-matching in the allocation algorithm.

This analysis is not devoid of limitation. To exclude center-specific factors and enlarge cohort size, we performed a bicentric analysis and included 105 patients. This is a rather low sample size, but big sample sizes in ESP programs are rare. Due to its retrospective nature, we could not test our new risk model for ICU admission in a prospective, independent manner. Before extrapolating our results to other centers, an external validation of our risk model will be needed. For this reason, we encourage other transplantation centers to test our risk model to further enhance its validity. With a bigger cohort size, the confidence intervals for the risk factors BMI, CAD and hypertensive nephropathy will potentially be reduced. Currently, our risk model is an excellent search test, but has a rather low PPV and therefore cannot replace individual and local risk assessment in times of reduced ICU capacities during the SARS-CoV-2 pandemic.

## 5. Conclusions

The SARS-CoV-2 pandemic has impacted health care systems tremendously worldwide, making the deferral of elective and non-urgent surgical interventions necessary due to limited PPE and ICU capacities. To provide a valid risk assessment tool concerning the risk of ICU admission for old patients in the Eurotransplant Senior Program, we have identified a low BMI, coronary artery disease and hypertensive nephropathy as significant predictors for ICU admission. For this reason, each transplant center should always carefully discuss whether local ICU capacities allow high-risk KT or not.

## Figures and Tables

**Table 1:** Comparison of patient characteristics with or without ICU stay after kidney transplantation in the ESP program.

	∑ (*n* = 105)	ICU Yes (*n* = 17)	ICU No (*n* = 88)	*p*
**Recipient**				
age (year)	69 (65; 82)	71 (65; 80)	69 (65; 82)	n.s.
male gender	68 (64.8%)	10 (58.8%)	58 (65.9%)	n.s.
BMI (kg/m^2^)	26.3 (19.2; 37.9)	24.2 (19.3; 31)	26.7 (19.2; 37.9)	0.014
**Pre-transplant**				
hypertension	101 (96.2%)	17 (100%)	84 (95.5%)	n.s.
CAD	42 (40%)	11 (64.7%)	31 (35.2%)	0.031
diabetes	41 (39%)	6 (35.3%)	35 (39.8%)	n.s.
history of smoking	18 (17.1%)	2 (11.8%)	16 (18.2%)	n.s.
**Cause for ESKD**				
chronic GN	23 (18.9%)	1 (5.9%)	22 (25%)	n.s.
diabetic NP	17 (13.9%)	2 (11.8%)	15 (17%)	n.s.
hypertensive NP	15 (12.3%)	6 (35.3%)	9 (10.2%)	0.015
other *	50 (47.6%)	9 (52.9%)	46 (52.2%)	n.s.
time on dialysis (d)	918.5 (2; 3830)	1384 (484; 3830)	855.5 (12; 3302)	n.s.
hemodialysis	84 (80%)	16 (94.1%)	68 (77.3%)	n.s.
first Tx	101 (96.2%)	17 (100%)	84 (95.5%)	n.s.
**Graft**				
donor age (year)	71 (65; 85)	71 (66; 82)	71 (65; 85)	n.s.
HLA-mismatches	4 (1; 6)	4 (1; 6)	4 (1; 6)	n.s.
CIT (min)	571.8 (181.2; 1236)	667.8 (228; 1166.4)	552.3 (181.2; 1236)	0.053

* see Appendix Table A3 for further information.

**Table 2:** Perioperative outcome.

	∑ (*n* = 105)	ICU Yes (*n* = 17)	ICU No (*n* = 88)	*p*-Value
**Transplantation**				
operating time (min)	184 (116; 436)	212 (129; 268)	180 (116; 436)	n.s.
WIT (min)	46.5 (21; 126)	47 (35; 70)	46 (21; 126)	n.s.
complications	12 (11.4%)	2 (11.8%)	10 (11.4%)	n.s.
**Postoperative**				
complications				n.s.
none	42 (40%)	5 (29.4%)	37 (42%)	n.s.
minor	28 (26.7%)	3 (17.6%)	25 (28.4%)	n.s.
major	35 (33.3%)	9 (52.9%)	26 (29.5%)	n.s.
length of stay	19 (8–66)	21.5 (12–66)	18 (8–62)	n.s.
**Graft Function**				
DGF rate	42 (40%)	9 (52.9%)	33 (37.5%)	n.s.

**Table 3:** Age-dependent comparison stratifying for donor age (very old donors ≥75 years vs. old donors) or recipient age (very old recipients ≥70 years vs. old recipients).

	Donors: *Very Old*-For-Old vs. Old-For-Old			Recipients: Old-For-*Very* Old vs. Old-For-Old		
	Very Old (*n* = 28)	Old (*n* = 77)	*p*	Very Old (*n* = 47)	Old (*n* = 58)	*p*
**Transplantation**						
operating time	180 (120; 281)	188 (116; 436)	n.s.	190 (128; 268)	181 (116; 436)	n.s.
WIT (min)	46 (21; 126)	49.5 (32; 85)	n.s.	48 (32; 104)	46 (21; 126)	n.s.
complications	4 (14.3%)	8 (10.4%)	n.s.	6 (12.8%)	6 (10.3%)	n.s.
**Postoperative**						
ICU admission	6 (21.4%)	11 (14.3%)	n.s.	10 (21.3%)	7 (12.1%)	n.s.
Clavien–Dindo			n.s.			n.s.
none	13 (46.4%)	29 (37.7%)		16 (34%)	26 (44.8%)	
minor	10 (35.7%)	18 (23.4%)		13 (27.7%)	15 (25.9%)	
major	5 (17.9%)	30 (39%)		18 (38.3%)	17 (29.3%)	
length of stay	16 (12; 46)	20 (8; 66)	0.028	20 (10; 66)	18.5 (8; 65)	
**Graft Function**						
DGF	14 (50%)	28 (36.4%)	n.s.	19 (40.4%)	23 (39.7%)	n.s.

**Table 4:** Multivariate regression analysis to predict an ICU admission during the hospital stay.

Variable	OR (95% CI)	*p*-Value
BMI	0.80 (0.68; 0.94)	0.008
hypertensive nephropathy	4 (1.02; 15.67)	0.046
coronary artery disease	4.46 (1.32; 15.07)	0.016

**Table 5:** Significant impact factors on graft loss and patient death in multivariate cox regression.

Variable	HR (95% CI)	*p*-Value
**Graft Loss**		
number of Tx	9.66 (2.48; 37.69)	0.001
HLA-mismatches	1.53 (1.03; 2.27)	0.033
serum creatinine 1 mo	1.37 (1.01; 1.87)	0.04
**Patient Death**		
pre-transplant diabetes	2.22 (1.02; 4.86)	0.046
ICU admission	4.72 (2.02; 11.03)	<0.001

**Table 6:** Comparison of death-censored graft and patient survival in ESP programs.

	Frei [19] *n* = 1406	Quast [20] *n* = 217	Bahde [23] *n* = 89	Jacobi [21] *n* = 89	Our Results *n* = 105
**Graft Survival**					
1-year	75%	76.4% ^1^	n.a.	87%	84%
5-year	47%	57.3% ^1^	77%	63%	73%
**Patient Survival**					
1-year	86%	88.2% ^1^	n.a.	87%	85%
5-year	60%	71.8% ^1^	69.8%	63%	62%

^1^ Only considering old, but not very old, donors.

## Appendix A

### Appendix A.1. Risk Model for ICU Admission

In a multivariate binary logistic regression analysis, BMI, hypertensive nephropathy and coronary artery disease had significant impact on ICU admission. The prediction probability (P) of an ICU stay for each individual patient was calculated with the equation

$$P=\frac{1}{1+e^{-z}}$$

in which the logit *z* is

z=3.557+4.004×HN+1.495×CAD−0.221×BMI

- HN: presence of hypertensive nephropathy (binary: no = 0, yes = 1)
- CAD: presence of coronary artery disease (binary: no = 0, yes = 1)
- BMI: body-mass-index in kg/m^2^ (continuous)

The optimal cut-off for the predicted probability of ICU admission was calculated via ROC analysis by using a Youden index (see Figure A1). By setting the cut-off to 0.08, this risk model gained a sensitivity of 94.1%, specificity of 51.1%, false positive rate of 48.9%, false negative rate of 5.9%, positive predictive value of 27.1% and negative predictive value of 97.8% (see Table A1).

**Table A1:** Crosstabulation illustrating case assignment in our cohort by risk model.

	ICU Yes	ICU No	∑
risk model: ICU yes	16	43	59
risk model: ICU no	1	45	46
**∑**	17	88	105

### Appendix A.2. Graft and Patient Survival Stratified for Donor and Recipient Age

**Table A2:** Mortality table with age-dependent comparison stratified for donor age (very old donors ≥75 vs. old donors) or recipient age (very old recipients ≥70 years vs. old recipients).

	Donors: *Very* Old-For-Old vs. Old-For-Old			Recipients: Old-For-*Very* Old vs. Old-For-Old		
	Very Old (*n* = 28)	Old (*n* = 77)	*p*	Very Old (*n* = 47)	Old (*n* = 58)	*p*
**Graft Survival**			n.s.			n.s.
1 year	22 (78%)	61 (86%)		37 (87%)	46 (82%)	
5 years	11 (78%)	25 (72%)		18 (73%)	18 (75%)	
9 years	2 (58%)	6 (41%)		108 (60%)	4 (35%)	
**Patient Survival**			n.s.			n.s.
1 year	25 (78%)	63 (90%)		39 (82%)	49 (87%)	
5 years	12 (74%)	28 (59%)		21 (60%)	19 (63%)	
9 years	1 (36%)	6 (54%)		2 (55%)	5 (33%)	

### Appendix A.3. Underlying Renal Diseases

**Table A3:** Underlying renal diseases for patients with or without ICU stay after KT.

	∑ (*n* = 105)	ICU Yes (*n* = 17)	ICU No (*n* = 88)	*p*-Value
ADPKD	11 (10.5%)	2 (11.8%)	9 (10.2%)	n.s.
amyloidosis	3 (2.9%)	-	3 (3.4%)	n.s.
analgesic nephropathy	3 (2.9%)	1 (5.9%)	2 (2.3%)	n.s.
chronic glomerulonephritis	23 (21.9%)	1 (5.9%)	22 (25%)	n.s.
cardiac cirrhosis	1 (1%)		1 (1.1%)	n.s.
diabetic nephropathy	17 (16.2%)	2 (11.8%)	15 (17%)	n.s.
FSGS	2 (1.9%)	-	2 (2.3%)	n.s.
goodpasture syndrome	2 (1.9%)	-	2 (2.3%)	n.s.
hypertensive nephropathy	15 (14.3%)	6 (35.3%)	9 (10.2%)	<0.05
IgA nephropathy	3 (2.9%)	-	3 (3.4%)	n.s.
kidney cirrhosis	8 (7.6%)	2 (11.8%)	6 (6.8%)	n.s.
nephrosclerosis	7 (6.7%)	2 (11.8%)	5 (5.7%)	n.s.
other cystic disease	3 (2.9%)	1 (5.9%)	2 (2.2%)	n.s.
renal cell carcinoma	2 (1.9%)	1 (5.9%)	1 (1.1%)	n.s.
vascular nephropathy	7 (6.7%)	3 (17.6%)	4 (4.5%)	n.s.
vasculitis	2 (1.9%)	1 (5.9%)	1 (1.1%)	n.s.
not known	13 (12.5%)	-	13 (14.8%)	n.s.
